# Infectivity and transmissibility of an avian H3N1 influenza virus in pigs

**DOI:** 10.1186/s13567-022-01133-x

**Published:** 2023-01-24

**Authors:** Wojciech Stadejek, Koen Chiers, Kristien Van Reeth

**Affiliations:** 1 Laboratory of Virology, Department of Translational Physiology, Infectiology and Public Health, Faculty of Veterinary Medicine, Ghent University, Salisburylaan 133, 9820 Merelbeke, Belgium; 2 Laboratory of Veterinary Pathology, Faculty of Veterinary Medicine, Ghent University, Salisburylaan 133, 9820 Merelbeke, Belgium

**Keywords:** Avian influenza, swine, explants, H3N1, replication, transmission

## Abstract

In 2019 a low pathogenic H3N1 avian influenza virus (AIV) caused an outbreak in Belgian poultry farms, characterized by an unusually high mortality in chickens. Influenza A viruses of the H1 and H3 subtype can infect pigs and become established in swine populations. Therefore, the H3N1 epizootic raised concern about AIV transmission to pigs and from pigs to humans. Here, we assessed the replication efficiency of this virus in explants of the porcine respiratory tract and in pigs, using virus titration and/or RT-qPCR. We also examined transmission from directly, intranasally inoculated pigs to contact pigs. The H3N1 AIV replicated to moderate titers in explants of the bronchioles and lungs, but not in the nasal mucosa or trachea. In the pig infection study, infectious virus was only detected in a few lung samples collected between 1 and 3 days post-inoculation. Virus titers were between 1.7 and 4.8 log_10_ TCID_50_. In line with the ex vivo experiment, no virus was isolated from the upper respiratory tract of pigs. In the transmission experiment, we could not detect virus transmission from directly inoculated to contact pigs. An increase in serum antibody titers was observed only in the inoculated pigs. We conclude that the porcine respiratory tract tissue explants can be a useful tool to assess the replication efficiency of AIVs in pigs. The H3N1 AIV examined here is unlikely to pose a risk to swine populations. However, continuous risk assessment studies of emerging AIVs in pigs are necessary, since different virus strains will have different genotypic and phenotypic traits.

## Introduction

Influenza is a disease of birds and mammals that is caused by influenza viruses from the *Orthomyxoviridae* family. The influenza viruses are classified into types A, B, C and D, with influenza A viruses (IAVs) having the widest host range. IAVs are further classified into subtypes, based on the antigenic characteristics of the surface proteins hemagglutinin (HA) and neuraminidase (NA). Almost all HA (H1-16) and NA (N1-9) subtypes are found in aquatic wild birds, whereas mammals are susceptible to a limited number of HxNy combinations [1].

Wild aquatic birds are a natural IAV reservoir, and they transmit avian influenza viruses (AIVs) to poultry. Swine are natural hosts for IAVs of the H1N1, H1N2 and H3N2 subtype [2], which are endemic in swine populations worldwide. They are the most important mammalian species in the ecology of IAVs and there is occasional, bidirectional transmission of IAVs between humans and swine. In addition, swine are susceptible to infection with AIVs of several subtypes under experimental [3–7] and natural conditions [8–13].

Pigs are also potential “mixing vessels” in which avian and swine or human viruses can undergo reassortment, i.e., exchange their gene segments, creating a virus with a novel genotype [3, 14–16]. Three of the four known pandemic IAVs were reassortants and at least one originated in swine. Indeed, the 2009 pandemic H1N1 IAV originated from pigs and was a quadruple reassortant of two swine, one avian and one human IAV, highlighting the role of pigs as a source of zoonotic influenza viruses [17, 18].

The 1957 and 1968 pandemic IAVs were of the H2N2 and H3N2 subtype respectively. Those viruses were reassortants between the then circulating human seasonal influenza viruses and an avian IAV that donated the novel HA subtype [18].

While the host in which the 1968 reassortant emerged remains obscure, pigs are susceptible to infection with AIVs of the H3 subtype. In 2001 an H3N3 AIV was isolated in Canada from a pig with signs of a respiratory infection [10]. In a study by De Vleeschauwer et al. pigs were intranasally inoculated with two different mallard H3N8 isolates. These viruses were excreted nasally by pigs for 2 to 5 days [19]. Similar observations were made after intranasal inoculation of two pigs with a duck H3N2 isolate. In this instance the virus was shed for 12 h to 5 days [20]. In a study performed by Kida et al., pigs were inoculated with 38 AIVs of subtypes H2 to H13, including eight H3 isolates. Most of these AIVs of different subtypes caused a productive infection and were detected in the nasal swabs for 4 to 7 days, but four out of eight H3 viruses remained undetectable [3].

In 2019 an AIV epizootic occurred in Belgium. The disease was caused by an H3N1 virus, which was transmitted from wild aquatic birds and adapted to poultry and subsequently affected 82 layer, broiler and turkey farms. An unusually high mortality of up to 40% in laying hens and up to 100% reduction in egg laying rate was observed [21]. The abnormally high pathogenicity was associated with the utilization of plasminogen for HA proteolytic cleavage. Typically, low pathogenic AIVs ae dependent on trypsin-like proteases for HA cleavage and subsequent host cell entry, which limits their tropism to tissues in which these proteases are present. The NA of H3N1 is able to recruit plasminogen for HA cleavage, facilitating systemic spread of the virus [22]. This H3N1 outbreak occurred in an area with a dense swine population. Therefore, there was concern about potential transmission of the H3N1 virus from poultry to pigs and further spread in the swine population. In this study, we aimed to evaluate the potential risk for such a spillover by infection experiments in explants of the porcine respiratory tract and in pigs, and by a transmission experiment in pigs.

## Materials and methods

### Viruses

A/chicken/Belgium/136/2019 H3N1 (chB19) was isolated in embryonated chicken eggs from a trachea of a cock exhibiting neurological signs. The virus was passaged once in embryonated chicken eggs. The full genome sequence was determined by Oxford Nanopore Technologies MinION sequencing (accession numbers OP765329, OP765352, OP765398, OP417305, OP765492, OP765503, OP765504, OP765508). Sequence analysis, performed in MEGA X (version 10.1.7), showed a deletion in the stalk of the NA and a mutation in the NA that is associated with plasminogen recruitment [21, 22].

A/swine/Missouri/A01410819/2014 H3N1 (swMO14), a representative of North American swine-adapted IAVs of the same subtype was used as a positive control in the porcine respiratory tract explant infection experiment. The virus was obtained from the United States Department of Agriculture (USDA) and passaged once in Madin Darby Canine Kidney (MDCK) cells.

### Animals

For ex vivo and in vivo experiments, two and 26 4-week-old pigs were purchased from a high-health, IAV-negative commercial farm, respectively. The pigs were housed in HEPA-filtered animal units with ad libitum access to food and water for 7 days before the start of the experiments. To confirm their influenza-negative status, serum samples were tested in hemagglutination inhibition (HI) assays against A/sw/Belgium/1/98 (H1N1), A/sw/Gent/172/08 (H3N2), A/sw/Gent/7625/99 (H1N2) and the pandemic A/California/04/09 (pH1N1) virus strain.

### Ex vivo replication of chB19 in porcine respiratory tract explants

To assess the ability of chB19 to replicate in swine respiratory tract tissues, ex vivo inoculation of porcine respiratory tract explants was performed. Nasal (NE), tracheal (TE), bronchial (BE) and lung (LE) explants were collected as previously described [23] from two 4-week-old IAV-negative pigs. Pigs were euthanized by an injection of an overdose of 20% sodium pentobarbital in the jugular vein and subsequent bleeding.

Briefly, the nose was sawn off and cut to open the nasal cavity. The nasal mucosa was detached from the septum and the turbinates, cut into squares of 25 mm^2^. The trachea was divided into two parts by a vertical cut. The tracheal mucosa was separated from the underlying cartilage. The mucosal membranes were placed on a steel mesh at the air-liquid interface in 2 mL of NTE medium (100 U/mL penicillin, 0.1 mL/mL streptomycin, 0.1 mg/mL gentamycin, RPMI + GlutaMAX and DMEM + GlutaMAX in 1:1 ratio).

Lungs were separated into right and left lobe. The lung tissue of the left lobe was mechanically separated from the bronchi. Bronchial sections 3 mm long and 2 mm in diameter were cut and placed in glass culture tubes filled with 1 mL of BE medium (100 U/ mL penicillin, 0.1 mL/mL streptomycin, 1 µg/mL kanamycin, 0.02 M/100 mL HEPES, MEM + GlutaMAX). The tubes were placed on a slowly rotating device (0.5 turn/min).

The right lung was filled with 1% low-gelling-temperature agarose and placed at 4 °C until the agarose set. The lung was then cut into 25 mm^2^ squares and submerged in 1 mL LE medium (100 U/mL penicillin, 0.1 mL/mL streptomycin, 0.1 mg/mL gentamycin, 2.5 µg/mL insulin, 0.5 µg/mL retinol, 0.5 µg/mL hydrocortisone, D-MEM + GlutaMAX).

All explants were incubated at 37 °C. At 24 h post-culture (hpc) NE, TE and LE were transferred to 24-well plates and washed with phosphate-buffered saline (PBS). Inoculations were performed in triplicates by submerging the explants in 600 µL of respective medium containing 10^6^ TCID_50_ of chB19 or swMO14 for one hour at 37 °C. Afterwards, the explants were washed 3 times with PBS and transferred to culture plates. Inoculation and subsequent washing of BE was performed in glass culture tubes. Supernatant samples for virus titrations were collected at 1, 24 and 48 h post-inoculation (hpi) and stored at −70 °C until used.

Virus titrations were performed in MDCK cells in 96-well plates and followed by immunoperoxidase monolayer assay (IPMA). Supernatant samples were serially diluted 1:10 and 50 µL of each dilution was added into the wells with confluent MDCK cells. Plates were incubated at 37 °C and 5% CO_2_ for 5 days. The cells were then fixed with 4% paraformaldehyde for 15 min, incubated with mouse anti-influenza nucleoprotein monoclonal HB-65 antibodies (1:50 dilution) for 2 h and then incubated with goat anti-mouse horseradish peroxidase-conjugated antibodies (DAKO; 1:1000 dilution). Infected cells were visualized with hydrogen peroxide and 3-amino-9-ethylcarbazole (AEC) and the number of wells with visible infection was determined by light microscopy. The virus titers, expressed as log_10_ TCID_50_/mL were calculated as described by Reed and Muench [24].

### Pig infection experiment

To determine the replication efficiency of chB19, 12 pigs were inoculated intranasally. The inoculum consisted of 10^7^ EID_50_ of chB19 in 3 mL of PBS and was delivered intranasally with a 15 mm cannula attached to a syringe, 1.5 mL per nostril. Two pigs that served as negative controls were housed in a separate unit and were not inoculated.

Each day, from 1 to 6 days post-inoculation (dpi), two pigs were euthanized, as described above. The two negative control pigs were euthanized at the end of the experiment.

At necropsy, gross pathological examinations were performed as previously described [25].

The following samples of the respiratory tract were collected for virus detection: nasal mucosa (respiratory and olfactory part), nasopharynx, soft palate, tonsil, trachea (proximal and distal part), the apical, cardiac and diaphragmatic lobes of the right and left lung halves. Samples of the apical and cardiac lobes were pooled.

The samples of nasal mucosa respiratory part, proximal part of trachea and right cardiac lung lobe were processed for histopathological analysis. The tissue samples were placed in 4% formalin, embedded in paraffin, cut into 5 μm slices, stained with hematoxylin and eosin, and examined by a pathologist for the presence of microscopic lesions. The tissues were given a score, based on a paper by Balzli et al. [6]: 0—normal tissue; 1—minimal inflammation, slight infiltrate and edema; 2—focal inflammation, slight cellular debris, edema, infiltrate; 3—multifocal inflammation, mild necrosis and cellular debris, moderate edema and infiltrate; 4—diffuse inflammation, interstitial infiltrate, cellular debris, necrosis.

10% (w/v) tissue homogenates were prepared from nasal mucosa olfactory part, nasopharynx, and soft palate and 20% (w/v) tissue homogenates were prepared from all other samples in PBS supplemented with 10 IU/mL penicillin and 10 µg/mL streptomycin. The homogenates were stored at −70 °C until used in titration assay and RNA isolation. Virus titration assay was performed in MDCK cells as described above. To perform RT-qPCR, viral RNA was isolated from 200 µL of the tissue homogenates using INDICAL BIOSIENCE IndiSpin Pathogen Kit, according to the manufacturer’s instructions.

RT-qPCR was performed using a previously published set of primers and a probe targeting IAV M gene (IVA-M1-F (AGA TGA GTC TTC TAA CCG AGG TCG); IVA-M1.1R (TGC AAA AAC ATC TTC AAG TYT CTG); IVA-M1.2-R (TGC AAA GAC ACT TTC CAG TCT CTG) IVA-M1-FAM (FAM-TCA GGC CCC CTC AAA GCC GA-TAMRA)) [26, 27].

Each reaction mixture contained 1000 nM IVA-M1-F primer, 750 nM IVA-M1.1R and IVA-M1.2-R primers, 125 nM IVA-M1-FAM probe, 12.5 µL AgPath-ID One-Step RT-PCR buffer and 1 µL RT-PCR Enzyme Mix (Thermo Fisher Scientific), 400 µM of each dNTP and 5 µL RNA in total volume of 25 µL.

The RT-qPCR reaction used the following program: 45 °C for 10 min, 95 °C for 10 min, 45 cycles of denaturation at 95 °C for 15 s, annealing at 56 °C for 20 s and extension at 72 °C for 30 s.

Samples with Ct values > 37 were considered as negative.

### Pig transmission experiment

The transmissibility of chB19 in pigs was evaluated as previously described [19]. Six pigs were inoculated intranasally with chB19 as described above, and six contact pigs were introduced into the unit with the directly inoculated pigs at 2 dpi.

Clinical examination of pigs was performed daily from 7 days before the start of the experiment until 14 dpi. Following clinical sings were monitored: depression/lethargy, conjunctivitis, nasal discharge, sneezing, coughing, tachypnea, dyspnea, abdominal breathing, and rectal temperature [28].

Nasal swabs were collected to assess the viral shedding. Two rayon-tipped nasal swabs (one per nostril) were used to collect the nasal secretions from each pig daily from 0 dpi/0 days post-contact (dpc) until 14 dpi/12 dpc. The two swabs from each pig were combined and placed in 1 mL of transport medium (10% fetal calf serum, 100 IU/mL penicillin and 100 µg/mL streptomycin, 0.1 mg/mL gentamycin in PBS), shaken for 1 h and stored at −70 °C until used in titration assay as described above. RNA was isolated from nasal swabs samples and analyzed by RT-qPCR as described above. Blood samples for serological analyses were collected from the jugular vein at 0, 16/14, 23/21, 30/28 dpi/dpc from the directly inoculated and contact groups, respectively.

Serum antibody titers against chB19 were assessed in HI [29] and virus neutralization assays (VN) assays. The serum samples were heat-inactivated for 30 min at 56 °C. Prior to the HI assay, sera were incubated for 18 h at room temperature with receptor-destroying enzyme (RDE). The RDE was then inactivated with sodium citrate at 56 °C for 30 min. The serum samples were pretreated with horse red blood cells (hRBC) to remove non-specific agglutinins, diluted two-fold in PBS and mixed with 4 hemagglutinating units of chB19. After 1 h of incubation 0.5% hRBC were added to each well and observed for the presence of hemagglutination.

The VN assay was performed as previously described [30] by incubating twofold dilutions of the sera with 100 TCID_50_ of MDCK-grown chB19 virus. After 1 h, MDCK cells at concentration of 8 × 10^5^ cells/mL were added to the virus-serum mixture, incubated for 24 h at 37 °C and subsequently fixed with 4% paraformaldehyde. Virus-positive cells were visualized by IPMA staining as described above.

### Statistical analysis

Differences in virus titers in explants between chB19 and swMO14 were analyzed using two-way ANOVA with area-under-curve (AUC) as the outcome and adjusting for individual pigs. AUC was calculated using an assumed value of 0 TCID_50_/mL at 0 hpi and the observed data at timepoints 24 and 48 hpi. Analysis was performed in R (version 4.2.1).

## Results

### Ex vivo replication of A/Chicken/Belgium/136/2019 (chB19) is inefficient and restricted to bronchial and lung explants

**Figure 1:**
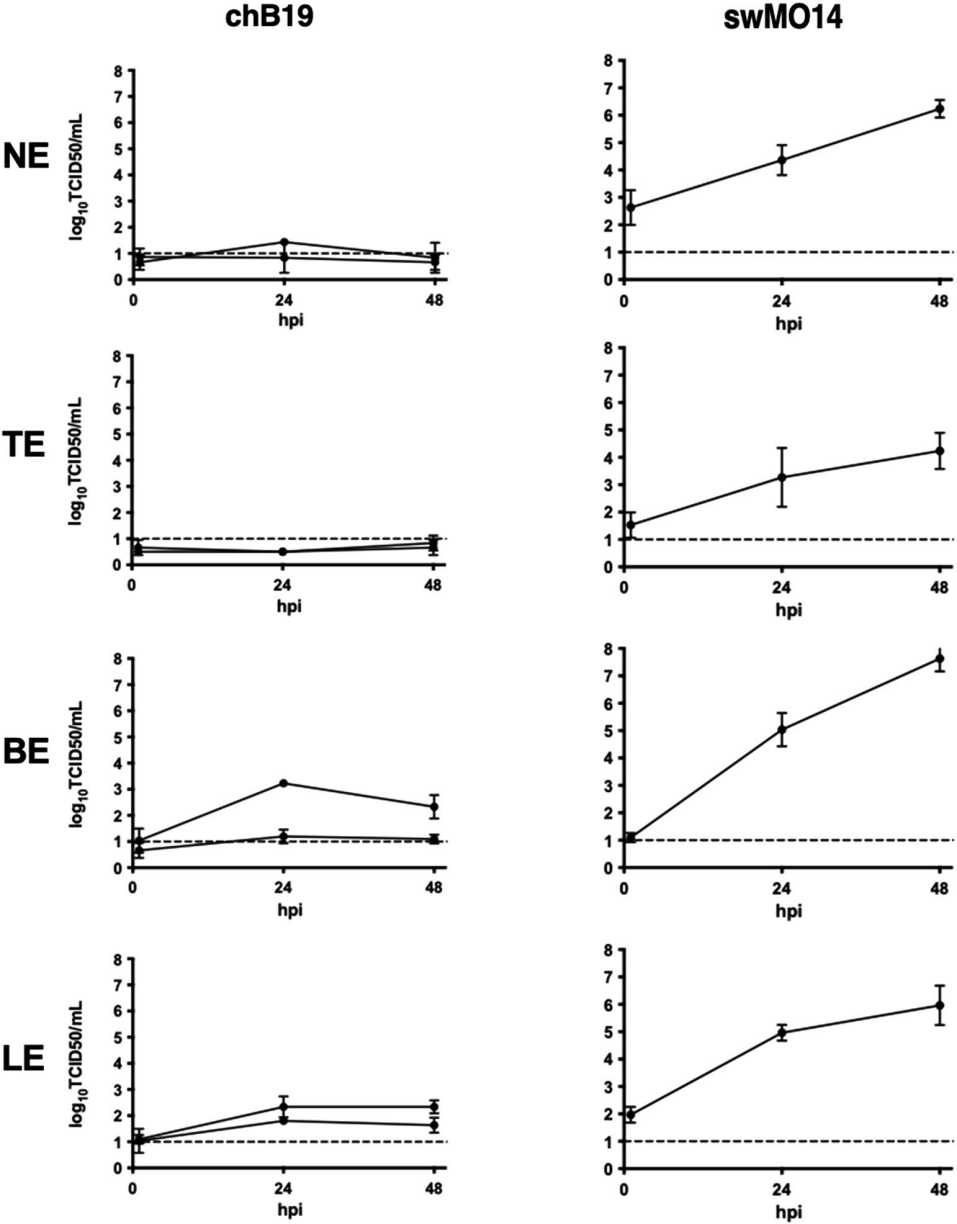
**Comparison of virus titers, expressed as log**_**10**_ **TCID**_**50**_**/mL, of A/chicken/Belgium/136/2019 (chB19) and A/swine/Missouri/A01410819/2014 (swMO14) in supernatant collected from inoculated nasal (NE), tracheal (TE), bronchial (BE) and lung (LE) explants at 1-, 24- and 48-hpi.** Each value represents a mean virus titer of three replicates and bars represent the standard deviation. The chB19 experiment was performed twice with explants collected from two different pigs. The dashed line represents the detection limit (1 log_10_ TCID_50_/mL)

The swine IAV A/swine/Missouri/A01410819/2014 (swMO14), used as a positive control, replicated efficiently in all four explant systems, with increasing virus titers until 48 hpi (Figure 1).

In contrast, infectious chB19 virus was barely detectable in nasal and tracheal explants, with virus titers below or close to the detection limit. An increase in chB19 virus titers was observed in bronchial explants, between 1 and 24 hpi, followed by a decrease between 24 and 48 hpi. Similarly, the titers in lung explants peaked at 24 hpi and did not increase further between 24 and 48 hpi. In both bronchial and lung explants, the chB19 titers were significantly lower than swMO14 titers (*p* < 0.0001).

### chB19 replicates poorly and restrictedly in the lower respiratory tract of intranasally infected pigs

To assess the replication efficiency and organ tropism of chB19 in pigs, 12 pigs were inoculated intranasally with a dose of 10^7^ TCID_50_. Two animals were euthanized daily from 1 to 6 dpi and samples of the upper and lower respiratory tract were collected for histopathological evaluation and virus quantification. Upon euthanasia, no gross lung lesions were observed in any of the chB19-inoculated pigs.

**Figure 2:**
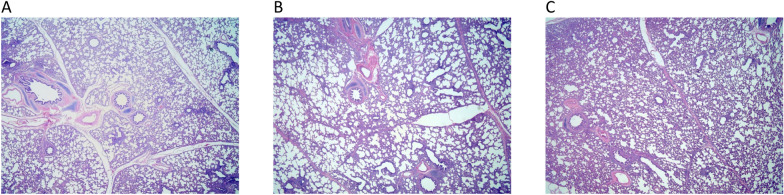
**Microscopic lung lesions in chB19-inoculatedpigs with detectable virus (A), undetectable virus (B) and uninoculated control pigs (C)**. Inflammatory infiltration and slight cellular debris are seen in all groups. The extent of lesions in chB19-inoculated pigs never exceeded that of control pigs

chB19 could not be detected by titration assay in MDCK cells from any of the 60 upper respiratory tract samples (Table 1; Figure 2). By contrast, six out of 72 (8.3%) lower respiratory tract samples were chB19-positive. The virus was isolated from two out of four (50%) lung samples of both pigs euthanized at 1 dpi, and from one out of four lung samples of one of both pigs euthanized on 2 and 3 dpi. No infectious virus was detected at 4, 5 and 6 dpi.

**Table 1:** **Detection of chB19 in the different parts of the swine respiratory tract by virus titration assay in MDCK cells (first line) and RT-qPCR (second line)**

Tissue	Titer (log_10_ TCID_50_/g tissue) Threshold cycle (Ct)											
	1 dpi		2 dpi		3 dpi		4 dpi		5 dpi		6 dpi	
	#1	#2	#3	#4	#5	#6	#7	#8	#9	#10	#11	#12
Nasal mucosa	<^a^	<	<	<	<	<	<	<	<	<	<	<
respiratory part	33.4	35.9	neg^b^	neg	neg	neg	32.5	neg	neg	36	neg	neg
Nasal mucosa	<	<	<	<	<	<	<	<	<	<	<	<
olfactory part^c^	neg	neg	neg	neg	neg	neg	neg	neg	neg	34.9	neg	neg
Nasopharynx^c^	<	<	<	<	<	<	<	<	<	<	<	<
	neg	34	neg	neg	neg	neg	36.1	neg	neg	neg	neg	neg
Soft palate^c^	<	<	<	<	<	<	<	<	<	<	<	<
	neg	neg	neg	neg	neg	neg	neg	neg	neg	neg	neg	neg
Tonsil	<	<	<	<	<	<	<	<	<	<	<	<
	neg	neg	neg	neg	neg	neg	neg	neg	neg	neg	neg	neg
Proximal trachea	<	<	<	<	<	<	<	<	<	<	<	<
	34.3	31.4	29.5	neg	neg	29.1	neg	neg	neg	neg	neg	neg
Distal trachea	<	<	<	<	<	<	<	<	<	<	<	<
	32.5	32.8	32.6	neg	36.5	30.5	neg	neg	neg	neg	neg	neg
Lung rightapical + cardiac lobes	**4.8**	**4.7**	**1.7**	<	**1.7**	<	<	<	<	<	<	<
	**18**	**17.5**	**21.8**	22.8	**25**	25.7	neg	27.2	31.5	29.7	32.3	32.2
Lung rightdiaphragmatic lobe	**1.7**	<	<	<	<	<	<	<	<	<	<	<
	**23.8**	35	26.4	29.4	neg	neg	neg	32.6	neg	neg	neg	neg
Lung leftapical + cardiac lobes	<	**4.3**	<	<	<	<	<	<	<	<	<	<
	35.6	**19.9**	25.8	26.3	25.7	28.8	34.8	neg	neg	33	neg	29.1
Lung right	<	<	<	<	<	<	<	<	<	<	<	<
diaphragmatic lobe	33.5	29	35.2	29.5	33.6	neg	neg	36.7	neg	neg	neg	neg

Samples positive by both RT-qPCR and titration are in bold.

^a^< Below the detection limit (1.7 log TCID_50_/g for 20% tissue homogenates, 2 log TCID_50_/g for 10% tissue homogenates).

^b^Result negative by RT-qPCR (Ct > 37).

^c^10% tissue homogenates were used for virus titration of these samples, 20% tissue homogenates were used for the remaining samples.

The tissue samples were also analyzed for the presence of viral RNA by reverse transcription quantitative polymerase chain reaction (RT-qPCR). Viral RNA was detected in 10% (6/60) of upper respiratory tract samples at relatively low amounts (Threshold cycle (Ct) 32.5–36, Table 1). Conversely, 54% (39/72) of lower respiratory tract samples were positive by RT-qPCR.

Histopathological analysis of negative control pigs showed minimal lesions in the nasal mucosa, no changes in the trachea and mild inflammation in the right cardiac lung lobe (Table 2). Of the 12 chB19-inoculated pigs, five exhibited minimal lesions in the nasal mucosa, two showed minimal lesions in the trachea and all pigs had minimal to mild inflammation, infiltrate, and edema in the right cardiac lung lobe.

**Table 2:** **Average microscopic lesion scores of selected respiratory tract tissues collected from chB19-inoculated pigs at different timepoints post-inoculation and from negative control pigs**

Tissue	Histopathological score*													
	1 dpi		2 dpi		3 dpi		4 dpi		5 dpi		6 dpi		Negative control	
	#1	#2	#3	#4	#5	#6	#7	#8	#9	#10	#11	#12	#13	#14
Nasal mucosa	0	1	1	0	0	1	0	0	1	0	1	0	1	1
Trachea	0	0	0	0	0	0	0	1	0	0	1	0	0	0
Right cardiac lung lobe	1	2	2	2	2	1	1	1	2	1	2	1	2	2

*Score and observed pathology:

0—Normal tissue

1—Minimal inflammation, slight infiltrate, and edema.

2—Focal inflammation, slight cellular debris, edema, infiltrate.

3—Multifocal inflammation, mild necrosis and cellular debris, moderate edema, and infiltrate.

4—Diffuse inflammation, interstitial infiltrate, cellular debris, necrosis.

In conclusion, chB19 replicates inefficiently in the porcine respiratory tract and mainly in the lungs.

### chB19 fails to transmit between pigs

To determine whether chB19 transmits between pigs, six pigs were inoculated intranasally with 10^7^ TCID_50_ of chB19 and cohoused with six naïve contact pigs 2 days later.

Virtually no clinical signs were observed in the chB19-inoculated pigs. From 2 dpi, four out of six animals experienced tachypnea. One pig showed fever (40.5 °C) at 3 and 4 dpi. Four pigs from the contact group showed tachypnea from 8 dpi until 13 dpi. No depression, dyspnea, labored breathing, or coughing was observed in any of the animals.

None of the pigs of the directly inoculated or contact group had detectable infectious virus in nasal swabs. Most directly inoculated pigs developed a serological response (Table 3). However, antibody titers remained ≤ 24 in the VN assay and they did not exceed the detection limit in the HI assay. No HI or VN antibodies were detected in contact pigs (data not shown).

**Table 3:** **Serological response against chB19 in directly inoculated pigs by virus neutralization (VN) and hemagglutination inhibition (HI) assay**

Assay	Number of seropositive pigs / total number of pigs (titer range)			
	0 dpi	16 dpi	23 dpi	30 dpi
VN	0/6 ( <4)	4/6 (12–24)	5/6 (8–24)	5/6 (12–16)
HI	0/6 ( <10)	4/6 (10)	4/6 (10)	1/6 (10)

## Discussion

The aim of this research was to determine the replication and transmission potential of the avian H3N1 virus strain chB19 in pigs. While the pigs were susceptible to the virus, replication was limited to bronchial and lung tissue explants ex vivo, and to the lungs in the pig infection study. chB19 failed to transmit from inoculated to contact pigs. An increase in antibody titers of inoculated pigs was observed, although only reaching low levels.

Only samples of the lungs collected from pigs on 1 to 3 dpi were positive in virus titration assays, while further analysis with RT-qPCR showed more positive samples, in upper respiratory tract tissues and in the lungs at later timepoints. This could mean that chB19 replicated in these tissues but to titers which are below the detection limit of the titration assay. It is also possible that fragments of viral RNA are present in the swine respiratory tract after 3 dpi, as the RT-qPCR used in this study cannot indicate whether the detected RNA comes from an infectious virus.

The chB19 tropism towards the lower respiratory tract can be explained by the receptor distribution in the swine respiratory tract and virus receptor binding preference. While the upper respiratory tract and trachea contain mostly Siaα2,6-galactose, the lungs contain both Siaα2,6- and Siaα2,3-galactose [23, 31]. Hemagglutinin of chB19 contains glutamine and glycine at positions 226 and 228 (226Q, 228G) respectively, which confers the binding preference towards avian-like Siaα2,3-galactose receptor [32, 33]. The introduction of 226Q and 228G mutations into the 1968 pandemic H3N2 virus decreased its replication efficacy in pigs, suggesting that Siaα2,3-galactose binding preference hinders AIVs to replicate in pigs [34].

Besides the receptor tropism, the viral RNA polymerase is another major determinant of AIV replication potential in pig tissues. Several mutations in a gene encoding the polymerase basic protein 2, such as E627K, D701N or G560S/Q591R in conjunction with T271, have been shown to increase AIV polymerase activity and thus virus replication in pig cells [35, 36]. The lack of these mutations in the PB2 gene of chB19 is likely a factor in the poor replication of chB19 in pig tissues.

Before the onset of the H3N1 epizootic, two notable NA gene mutations occurred: (1) the loss of a glycosylation site at amino acid position 130, allowing the recruitment of plasminogen and facilitating a systemic replication in chickens [22], and (2) a deletion in NA stalk. The latter feature is common after transmission of AIVs from waterfowl to poultry [37]. A shorter NA stalk is advantageous for the virus in chickens, as it enhances viral replication [38]. Simultaneously, a truncation of the NA may act as a barrier to infection of other hosts, such as ferrets. Blumenkrantz et al. created two influenza viruses containing genes encoding either truncated or full-length avian N1 and seven remaining genes of the 2009 pandemic H1N1 virus. Upon intranasal inoculation of ferrets, the full-length stalk virus spread to non-inoculated animals through both respiratory droplets and direct contact, while the short stalk reassortant did not transmit through respiratory droplets. This difference was associated with an inefficient mucus penetration and aggregation of virions [39]. It would be of interest to see whether the chB19 virus with a full-length NA stalk would be more replication-competent and transmissible between pigs.

The observed replication patterns of chB19 in the ex vivo respiratory tract explants were similar to those observed during the in vivo study. This suggests that explants might be routinely used to determine the potential of AIVs to infect pigs, and help to comply with the Three Rs principle (Replacement, Reduction, Refinement). However, more parallel pig and explant infection experiments with different AIV isolates would be required for more evidence.

The H3N1 AIV isolated from poultry that was used in this study does not pose a significant risk to a swine population. However, it cannot be ruled out that even a poorly replicating H3N1 virus can undergo reassortment in case of a coinfection of a pig with a swine influenza virus. It was shown that swine-adapted internal genes may increase the replication and transmission of AIVs in pigs [30, 40–42]. Further research on the potential effect of swine-adapted internal genes on the infectivity of the H3N1 virus will help understand how AIVs may cross the species barrier between birds and pigs.

The H3N1 AIV isolated from poultry that was used in this study virus does not pose a significant risk to a swine population. However, continuous risk assessment studies are needed to identify AIVs that could potentially infect and transmit between pigs. Explants might be a useful tool in assessment of the replication potential of AIVs in pigs, but they cannot replace transmission experiments.

## Data Availability

The data supporting the conclusions of this article are attached within the article.
